# Novel agents for pancreatic ductal adenocarcinoma: emerging therapeutics and future directions

**DOI:** 10.1186/s13045-017-0551-7

**Published:** 2018-01-31

**Authors:** Yiyin Zhang, Chao Yang, He Cheng, Zhiyao Fan, Qiuyi Huang, Yu Lu, Kun Fan, Guopei Luo, Kaizhou Jin, Zhengshi Wang, Chen Liu, Xianjun Yu

**Affiliations:** 1 Department of Pancreatic Surgery, Fudan University Shanghai Cancer Center, No. 270 DongAn Road, Shanghai, 200032 People’s Republic of China; 2 Department of Oncology, Shanghai Medical College, Fudan University, No. 270 DongAn Road, Shanghai, 200032 People’s Republic of China; 3 Shanghai Pancreatic Cancer Institute, No. 270 DongAn Road, Shanghai, 200032 People’s Republic of China; 4 Pancreatic Cancer Institute, Fudan University, No. 270 DongAn Road, Shanghai, 200032 People’s Republic of China

**Keywords:** Pancreatic ductal adenocarcinoma, Novel regimens, Biomarkers, Combination therapies

## Abstract

A poor prognosis of pancreatic ductal adenocarcinoma (PDAC) associated with chemoresistance has not changed for the past three decades. A multidisciplinary diagnosis followed by surgery and chemo(radiation)therapy is the main treatment approach. However, gemcitabine- and 5-fluorouracil-based therapies did not present satisfying outcomes. Novel regimens targeting pancreatic cancer cells, the tumor microenvironment, and immunosuppression are emerging. Biomarkers concerning the treatment outcome and patient selection are being discovered in preclinical or clinical studies. Combination therapies of classic chemotherapeutic drugs and novel agents or novel therapeutic combinations might bring hope to the dismal prognosis for PDAC patients.

## Background

Pancreatic ductal adenocarcinoma (PDAC) is associated with the poorest prognosis of gastrointestinal cancers and will become the second leading cause of cancer-related mortality in the USA by 2030. Only 10–20% of patients are resectable when first diagnosed [1]. An aggressive tumor biological nature, a desmoplastic microenvironment, and resistance to standard cytotoxic therapies also contribute to the poor prognosis.

It has been identified that KRAS, TP53, CDKN2A, and SMAD4 are four major driver genes participating in the whole process of disease development [2]. More than 90% of tumors harbor KRAS mutations, which are known to increase the tumor invasive ability by reprogramming pancreatic cell metabolism and promoting stromal reaction. Complex genetic and metabolic pathways were identified and utilized for treatment. Additionally, some receptors that are essential to certain pathways were studied for therapeutic use, including epidermal growth factor receptor (EGFR), human epidermal growth factor receptor 2 (HER2), and vascular endothelial growth factor receptor (VEGFR).

Jones S et al. first found 12 core signaling pathways in pancreatic cancer (PC) and determined that they were each genetically altered in 67 to 100% of the tumors by performing global genomic analysis [3]. Their finding suggested that targeting downstream mediators or key nodal points might be more effective than targeting specific mutated genes. Bailey et al. revealed that the complex mutational landscape of PDAC comprised four subtypes—squamous, pancreatic progenitor, immunogenic, and aberrantly differentiated endocrine exocrine (ADEX). Squamous, pancreatic progenitor, and ADEX tumors were enriched in different mutations, such as TP53 and KDM6A, FOXA2/3, PDX1, and MNX1, and expressed preferentially at the early stage and with regulative genes involved in KRAS activation. By targeting certain immune modulators, specific mechanisms underlying the immunogenic subtype inferred future therapeutic development. Ten distinct molecular mechanisms concerning 32 recurrently mutated genes were also identified and included KRAS, TGF-β, WNT, Notch, ROBO/SLIT signaling, G1/S transition, SWI-SNF, chromatin modification, DNA repair, and RNA processing [4]. Notta et al. found that this aggressive disease progressed rapidly as mitotic errors of complex rearrangement pattern occurred simultaneously rather than sequentially [5]; the tumor microenvironment plays a key role in treatment resistance because it creates a mechanical barrier consisting of dense stroma with fibroblasts, leukocytes, hyaluronic acid (HA), cancer stem cells (CSCs), collagen, and some extracellular matrix protein, resulting in hypoxia and anti-angiogenesis, which promotes carcinogenesis, facilitates tumor progression and induces chemotherapeutic resistance.

Great effort has been made to combine classic chemotherapies and novel agents targeting the discovered mutations and pathways to improve the outcome of PDAC.

### Current therapies for PDAC

The CONKO-001 trial showed that the addition of 6 months of gemcitabine as adjuvant therapy could prolong disease-free survival (DFS) and overall survival (OS) in patients with resected PC. The minimal survival benefit between the gemcitabine and observation groups could be explained by gemcitabine treatment after disease progression in the control arm (DFS, 13.4 vs 6.9 months, *P* < 0.001; mOS, 22.1 vs 20.2 months, *P* = 0.06) [6]. Gemcitabine-based chemotherapy is the current standard adjuvant therapy, as an 11-year follow-up study showed it dramatically decreased 45% of the DFS rate and 24% of the mortality risk [7]. The ESPAC-3 trial included 1088 patients after surgery to receive either bolus 5- fluorouracil (5-FU) followed by intravenous 5-FU or gemcitabine for 6 months. The median survival agreed with that in the CONKO-001 trial (23.0 vs 22.1 months), with no significant difference between the bolus of 5-FU and gemcitabine, but the gemcitabine arm showed a 10% improvement in OS [8]. Nab-paclitaxel and FOLFIRINOX (folinic acid, fluorouracil, irinotecan, oxaliplatin) showed considerable efficacy in the metastatic setting [9, 10]. Direct comparison of the efficacy in the adjuvant setting between nab-paclitaxel/gemcitabine and FOLFIRINOX for resected PDAC is still under clinical trials, including the APACT and PRODIGE trials (NCT01964430, NCT01526135). With the modern radiation technique updates, a remarkable promotion of survival was presented by higher dose radiotherapy plus chemotherapy in early-stage resected PDAC [11]. Thus, it raises hope that chemoradiation therapy might extend the survival in patients with PDAC.

Although approximately 20% of early-staged PDAC patients undergo resection, the 5-year survival rate remains low. Tagged cells of the PC mouse model unexpectedly entered the bloodstream and seeded into the liver without the invasion of primary lesions, suggesting that PDAC is a systemic disease rather than localized. Xu and Liu et al. discovered that patients with CEA(+)/CA125(+)/CA19–9 ≥ 1000 U/mL showed no survival advantage benefit from pancreatectomy and postoperative elevation of CA125/CEA with normal CA19–9 being associated with a poor prognosis [12, 13]. Luo et al. sequenced fucosyltransferase 3, pointing out that CEA and CA125 have the potential to be applied as biomarkers and should be routinely measured for Lewis-negative genotypes [14]. Several phase I/II retrospective studies showed that neoadjuvant therapy benefited patients with high-risk factors by promoting secondary resection rates, and FOLFIRINOX presented valuable efficacy. However, because phase III randomized control and multicenter clinical trials are lacking, the optimized regimen for neoadjuvant therapy has not yet been validated [15–20].

Gemcitabine-based therapy is the standard treatment for metastatic PDAC (MPC). Compared with gemcitabine, FOLFIRINOX showed a survival advantage for good-performance patients, and monotherapy S-1 presented well-tolerated, non-inferiority OS in patients with gemcitabine-refractory PDAC [10, 21]. Nab-paclitaxel/gemcitabine demonstrated an improved response rate (23%), progression-free survival (PFS) (5.5 months), OS (8.5 months), and a better HR of 0.72 compared with other phase III studies of gemcitabine-based therapy. Although erlotinib could decrease tumor growth, prevent metastasis, and improve the anticancer effect of gemcitabine to some extent, with only a 10-day benefit, it is not routinely applied in the clinical setting because the biomarkers for a treatment response are lacking despite skin rash and p53 expression [22]. The anti-EGFR monoclonal antibody cetuximab inhibits PC cell growth via antibody-dependent cell-mediated cytotoxicity and complement-dependent cytotoxicity, but a phase III study failed to prove a clinical benefit compared with gemcitabine (mOS, 6.3 vs 5.9 months, *P* = 0.23; mPFS, 3.4 vs 3.0 months) [23]. EGFR and the HER2 inhibitor lapatinib did not show obvious survival improvement in a single-arm phase II study (mPFS, 2.6 months, *P* = 0.001; mOS, 5.2 months, *P* = 0.023) [24]. Thus, the study indicated that EGFR/HER2 expression, quantified by immunohistochemistry, can hardly identify tumorigenetic subgroups driven by EGFR/HER2, and biomarkers predicting a good response have not yet been detected. Familial PDAC harbors BRCA mutations, which reduce gene recombination and DNA damage repair ability. PARPi inhibits the DNA repair of cancer cells and enhances the efficacy of platinum drugs. Olaparib monotherapy presented a promising response to the second-line setting because it showed a response rate of 20% and a median OS of 9.8 months. PARPi is the first real PC-targeted drug, and it has the potential to be used for combination therapy [25].

### Novel therapies in PDAC

#### Therapies targeting pancreatic cancer cells

##### Enhanced efficacy of cytotoxic agents

Because the efficacy of classic PDAC cytotoxic agents is limited, novel formulations of classic agents or drugs targeting the desmoplastic and hypoxic environment were developed. A randomized phase III study NAPOLI-1 added nanoliposome irinotecan prior to 5-FU/leucovorin (LV) in MPC, and the results showed that the objective response rate (ORR) and median OS were significantly improved compared with those in patients in the 5-FU/LV arm (mOS: 6.1 vs 4.2 months, *P* = 0.012) [26]. An oral prodrug of 5-FU, S-1, was verified as non-inferior, even superior, and was well tolerated compared with gemcitabine among resected PC patients from Asia in the JASPAC-01 study (HR = 0.57, 95% CI 0.44–0.72, *P* < 0.0001) [27]. This study was launched in 33 hospitals in Japan, and the proportion of male patients younger than 65 years old with a performance status (PS) of 0 was relatively larger in the S-1 group than in the gemcitabine group (male 57 vs 54%, < 65 years old 41 vs 39%, PS 0 70 vs 67%). Another oral prodrug of 5-FU, capecitabine, in combination with gemcitabine as adjuvant therapy among resected PC patients from Europe, showed a significantly better median OS than gemcitabine alone (28 vs 22.5 months, *P* = 0.032) [28]. The 5-year survival was 28.8% in patients treated with capecitabine plus gemcitabine in the ESPAC-4 trial compared with 44.1% in the S-1 group of the JASPAC-01 trial. The large survival benefit difference between the two trials might because the JASPAC trial included patients with a better risk, such as a lower R1 resection rate (13 vs 60%), a lower proportion of CA19–9 levels exceeding the upper normal limit (26 vs 32%), and a higher proportion of N0 patients (37% vs 20%) and PS0 patients (69 vs 42%). Oral prodrugs of 5-FU are considered new standard treatment for patients with resectable PC after surgery despite racial differences. Global multicenter phase III studies are needed to further confirm racial differences and possibly the underlying mechanism, indicating a possible breakthrough for Asia PDAC patients. Evofosfamide is a prodrug that selectively targets tumor cells under hypoxic conditions and results in the release of a strong alkylating agent, dibromoisophosphoramide mustard. A phase II trial of evofosfamide demonstrated a 2.4-month longer PFS for unresectable PDAC than in those treated with gemcitabine alone (*P* = 0.005) [29]. Based on the encouraging result of the phase II study, the phase III MAESTRO randomized trial of evofosfamide and gemcitabine as first-line therapy was recently completed (NCT01746979), showing an improved OS compared with gemcitabine alone (mOS, 8.7 vs 7.6 months, *P* = 0.059). However, because patients from Asia achieved a longer OS than other races, further confirmation of subgroup analysis could be a breakthrough for Asia PDAC patients.

##### RAS/RAF/MEK/ERK and PI3K/AKT/mTOR pathways

KRAS mediates signal transduction between membrane growth factor receptors and downstream pathways, such as PI3K/AKT/mTOR and RAS/RAF/MEK/ERK, which all contribute to tumor growth, progression, and metastasis [30]. Efforts have been made to target KRAS, but all have failed, such as the use of the farnesyltransferase inhibitor R115777 and tipifarnib [31, 32]. However, a new approach to target KRAS through engineered exosomes carrying short interfering RNA or short hairpin RNA specific to oncogenic *Kras*^*G12D*^ was recently discovered in a PC mouse model and proved to be associated with increased OS [33]. The use of engineered exosomes and interfering RNA offered a potential prospective for even more accurate transportation for precision treatment. Chu et al. found that KRAS-integrin-linked kinase (ILK) allows PC cells to regulate KRAS expression, and the inhibition of ILK blocked KRAS-driven epithelial-to-mesenchymal transition (EMT) as well as growth factor-stimulated KRAS expression. Thus, ILK inhibitors may be a viable therapeutic strategy for PDAC [34].

**Fig. 1:**
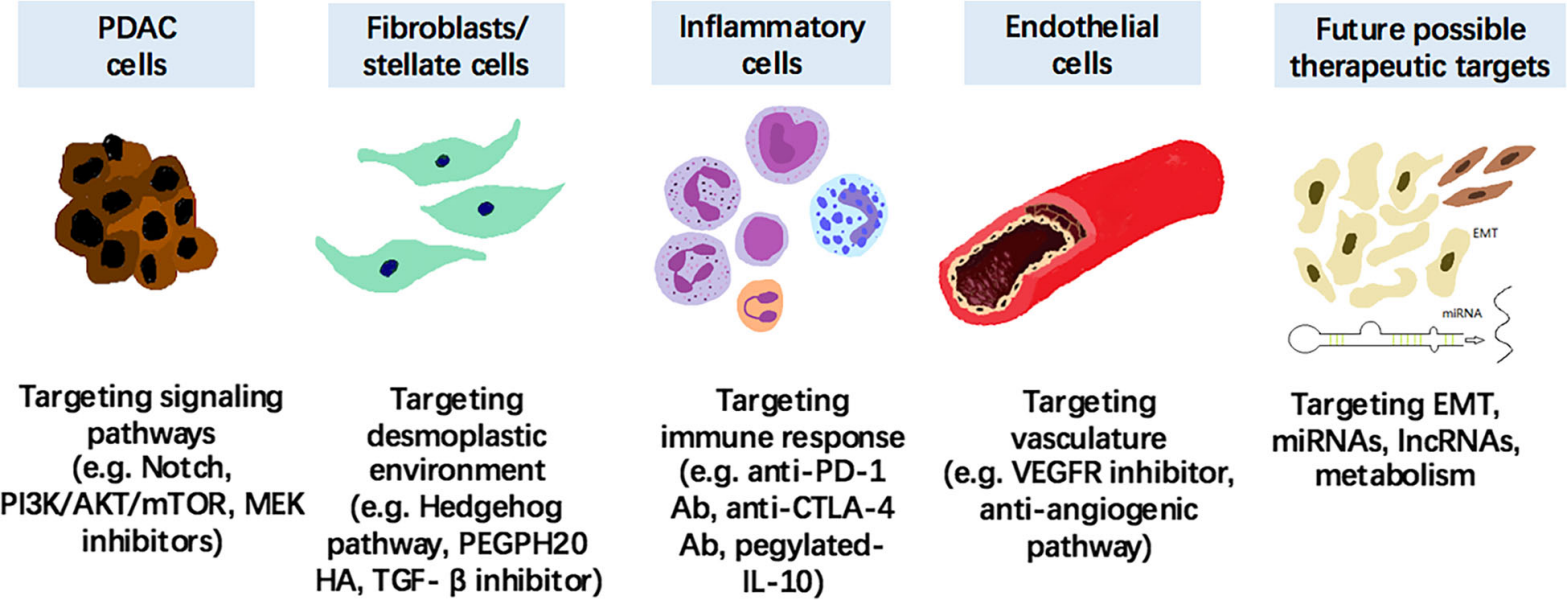
Novel therapies targeting different elements involved in PDAC development. Therapeutics targeting signaling pathways participating in tumorigenesis and progression, the tumor microenvironment, inflammatory and immune responses, and angiogenesis. It also contains future possible therapeutic targets and specific biomarkers for the evaluation of efficacy

**Fig. 2:**
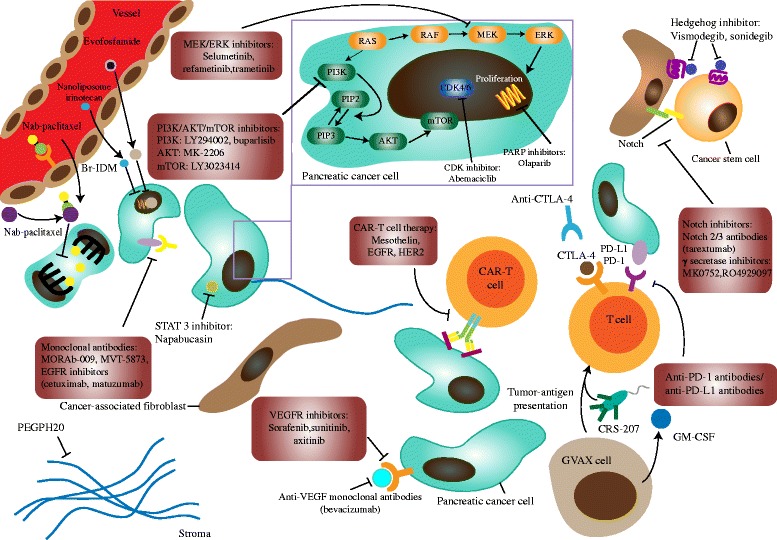
Mechanism of action of different therapeutic strategies for PDAC. They include cytotoxic agents, PARP inhibitors, CDK inhibitors, MEK/ERK inhibitors, PI3K/AKT/mTOR inhibitors, Hedgehog inhibitors, Notch inhibitors, STAT 3 inhibitors, VEGFR inhibitors, monoclonal antibodies, GVAX/CRS-207, anti-PD-1/PD-L1 antibodies, and CAR-T cell therapy

Other therapeutic strategies were mainly developed targeting the downstream effectors MEK, PI3K/AKT, and ERK. The PI3K-AKT-mTOR signaling pathway regulates cell metabolism, cell cycle, protein synthesis, and apoptosis. The PI3K inhibitor LY294002 can inhibit cancer cell growth and suppress angiogenesis [35]. Drug combinations targeting multiple pathways might achieve a certain clinical benefit. However, the potent and highly selective MEK1/2 inhibitor selumetinib plus the PI3K/AKT inhibitor MK-2206 did not show a survival benefit but increased adverse events compared with mFOLFOX alone in gemcitabine-refractory MPC patients (Fig. 1) [36]. It is possible that the toxicity effects of two or more kinase inhibitors overlapped, and 45% of the patients in the experimental arm experienced toxicity-related treatment delays and dose reduction. Refametinib is an orally active, non-competitive MEK kinase inhibitor with high affinity to MEK 1/2. A continuous dosing regimen maximized refametinib-induced tumor growth inhibition. The half-life of refametinib was approximately 10–20 h after dosing on day 1, which was significantly longer than 8.3 h of selumetinib. Refametinib is generally well tolerated compared with selumetinib because grade 3–4 rash, and hematologic toxicity were observed more often in selumetinib users. The highly selective MEK1/2 inhibitor refametinib showed a promising ORR (ORR 23%; mOS, 8.9 months), similar to previously reported trametinib and gemcitabine (ORR 22%; mOS, 8.4 months) in advanced pancreatic cancer (APC) patients with detectable KRAS mutations (Fig. 2) [37, 38]. Because it showed a trend toward improved outcomes in APC patients without KRAS mutations (ORR 48%; mOS, 18.2 months), refametinib in combination with gemcitabine is especially encouraged when patients harbored KRAS mutations. MEK inhibitors showed disappointing efficacy as single agents because they often block the Ras-MARK pathway and activate PI3K together. The compromised efficacy might be due to the existence of resistance cells imposed by the MEK inhibitor and intratumoral heterogeneity [39]. A phase I study combining the PI3K inhibitor buparlisib with sonidegib or mFOLFOX6 was recently completed (NCT01571024). Moreover, cell cycle checkpoint inhibitors have been emerging in recent years and have showed considerable efficacy in reducing PC growth in preclinical study [40]. A phase II study of the PI3K/mTOR/DNA-PK inhibitor LY3023414 and CDK inhibitor abemaciclib is currently recruiting, and the results of this combination are awaited (NCT02981342, Table 1). Targeting the downstream effectors of KRAS mutation showed mediocre results, and more effort should be devoted to new approaches for targeting KRAS.

**Table 1:** Ongoing clinical trials of novel agents for PDAC

Category	ClinicalTrial.gov ID	Therapeutic agents	Mechanism of action	Study phase	Status	Treatment setting	Outcomes
Checkpoint inhibitors	NCT01473940	Gemcitabine hydrochloride + ipilimumab	Anti-CTLA-4 monoclonal antibody	I	Active, not recruiting	Recurrent PDAC	MTD, DLT, TTP, PFS, OS
	NCT02309177	Gemcitabine/carboplatin + nab-Paclitaxel + nivolumab	Anti-PD-1 antibody	I	Recruiting	PC	DLT, AE, PFS, OS, DCR, ORR, DoR
	NCT02558894	MEDI4736 ± tremelimumab	MEDI4736: anti-PD-L1 antibody; tremelimumab: anti-CTLA-4 antibody	II	Completed	MPC	ORR, DoR, PFS
	NCT02734160	Galunisertib + durvalumab	Galunisertib: TGF-β receptor inhibitor; durvalumab: anti-PD-L1 antibody	I	Recruiting	MPC	DLT, PFS, ORR, DoR, DCR, TTR, OS
	NCT02777710	Durvalumab + pexidartinib	Durvalumab: anti-PD-1 antibody; pexidartinib: CSF-1R, Flt3 and Kit inhibitor	I	Recruiting	MPC	DLT, ORR, DoR, PFS, AE
	NCT02879318	Gemcitabine + nab-paclitaxel ± durvalumab + tremelimumab	Durvalumab: anti-PD-L1 antibody; tremelimumab: anti-CTLA-4 antibody	II	Recruiting	MPC	OS, PFS, ORR, AE
Other immunotherapies	NCT01362790	SS1P + cyclophosphamide + pentostatin	Anti-mesothelin antibody	I/II	Ongoing, not recruiting	PC	ORR, AE, OS, PFS, DoR
	NCT01417000	GVAX + cyclophosphamide + CRS-207	GVAX (vaccine): irradiated GM-CSF of PDAC; CRS-207: listeria monocytogenes-expressing mesothelin	II	Completed	MPC	OS
	NCT02243371	GVAX + CRS-207 ± nivolumab	GVAX (vaccine): irradiated GM-CSF of PDAC; CRS-207: listeria monocytogenes-expressing mesothelin; nivolumab: anti-PD-1 antibody	II	Ongoing, not recruiting	MPC	OS, AE, PFS, TTP, ORR
	NCT02399137	MM141 + gemcitabine/nab-paclitaxel	MM141: bispecific antibodies	II	Ongoing, not recruiting	MPC	PFS, OS, ORR, DoR, AE
	NCT02587689	anti-MUC1 CAR-T cells	Autologous T cells	I/II	Recruiting	Resected PC	AE
	NCT02672917	MVT-5873	CA19–9 monoclonal antibody	I	Recruiting	PC	MTD, ORR, DoR, AE
	NCT02706782	Meso-CART	Mesothelin redirected autologous T cells	I	Recruiting	APC	AE
	NCT02713984	Anti-HER2 CAR-T	–	I/II	Recruiting	Refractory PC	AE, MTD
	NCT02923921	FOLFOX + AM0010	Pegylated-IL-10	III	Recruiting	MPC	OS, PFS, ORR
	NCT03018405	NKG2D CAR-T cells	–	I	Recruiting	Refractory PC	AE
Cancer stem cell therapy	NCT02231723	Gemcitabine + nab-paclitaxel/mFOLFIRINOX + BBI608	STAT3, Nanog, beta-catenin inhibitor	Ib	Ongoing, not recruiting	MPC	AE, DLT
	NCT02993731	Gemcitabine + nab-paclitaxel + napabucasin	STAT3 transcription inhibitor	III	Recruiting	MPC	OS, PFS, ORR, AE, DCR, QoL
Cytotoxic agents	NCT01746979	Gemcitabine + TH-302	Prodrug activated under hypoxic condition	III	Completed	LAPC/MPC	OS, PFS, DCR, QoL, AE
DNA repair	NCT01585805	Gemcitabine hydrochloride + veliparib	PARP inhibitor	II	Recruiting	LAPC/MPC	ORR, DCR, OS, PFS
	NCT02498613	Olaparib + cediranib	Olaparib: PARP inhibitor; cediranib: VEGFR inhibitor	II	Recruiting	Unresectable PC	ORR, AE, PFS
	NCT02890355	FOLFIRI/mFOLFIRI + veliparib	PARP inhibitor	II	Recruiting	MPC	OS, DCR, DoR, AE, ORR, PFS
Targeted therapies	NCT01571024	mFOLFOX6 + buparlisib	PI3K inhibitor	I	Completed	MPC	DLT, MTD, AE, PFS, OS
	NCT01647828	Gemcitabine + nab-paclitaxel + OMP-59R5	Anti-Notch2/3 Ab	Ib/II	Completed	MPC	DLT, MTD, PFS, OS, AE, ORR
	NCT02227940	Gemcitabine hydrochloride + cisplatin + ceritinib	Alk inhibitor	I	Recruiting	LAPC/MPC	MTD, AE, PFS
	NCT02349867	Gemcitabine + sorafenib + vorinostat + RT	Sorafenib: VEGFR, PDGFR, RAF/MEK/ERK inhibitor; vorinostat: HDAC inhibitor	I	Recruiting	PC	AE, PR, CR, PFS, OS, R0 resection rate
	NCT02495896	Gemcitabine hydrochloride/docetaxel/cisplatin/nab-paclitaxel + EphB4-HSA fusion protein	EshB4 inhibitor	I	Recruiting	Unresectable PC	DoR/SD, AE, OS, PFS
	NCT02671890	Gemcitabine hydrochloride + disulfiram	NF-κB inhibitor	I	Recruiting	MPC	MTD, AE, OS, ORR
	NCT02703571	Ribociclib + trametinib	Ribociclib: CDK 4/6 inhibitor; trametinib: MEK inhibitor	I/II	Recruiting	MPC	DLT, ORR, DoR, TTR, DCR, PDR, PFS, OS
	NCT02713529	AMG820 + pembrolizumab	AMG820: anti-CSF1R Ab; pembrolizumab: anti-PD-1 antibody	I/II	Recruiting	Advanced PC	AE, ORR
	NCT02981342	Abemaciclib + LY3023414 + gemcitabine/capecitabine	Abemaciclib: CDK inhibitor; LY3023414: PI3K/mTOR/DNA-PK inhibitor	II	Recruiting	MPC	DCR, PFS, DoR, OS
Stromal targeting	NCT01821729	FOLFIRINOX + losartan	Angiotensin-receptor blocker	II	Recruiting	LAPC	PFS, OS, AE, QoL
	NCT01839487	Gemcitabine + nab-paclitaxel a± PEGPH20	Pegylated recombinant hyaluronidase	II	Ongoing, not recruiting	MPC	PFS, AE, ORR, OS
	NCT02715804	Gemcitabine + nab-paclitaxel ± PEGPH20	Pegylated recombinant hyaluronidase	III	Recruiting	MPC	PFS, OS, ORR, DoR, AE
Metabolism	NCT01506973	Gemcitabine/abraxane + hydroxychloroquine	Autophagy inhibitor	I/II	Recruiting	MPC	OS
	NCT01835041	CPI-613 + mFOLFIRINOX	CPI-613: alpha-ketoglutarate dehydrogenase inhibitor	I	Ongoing, not recruiting	APC/MPC	MTD
	NCT02336087	Gemcitabine + nab-paclitaxel + metformin	–	I	Recruiting	Unresectable PC	DLT, AE, compliance, PFS, OS, TTF

*Ab* antibodies, *RT* radiation therapy, *MTD* maximum tolerated dose, *DLT* dose-limiting toxicities, *TTP* time to progression, *PFS* progression free survival, *OS* overall survival, *AE* adverse events, *DCR* disease control rate, *DoR* duration of response, *TTR* time to response, *CR* complete response, *PR* partial response, *SD* stable disease, *TTF* time to treatment failure, *PDR* progressive disease rate, *QoL* quality of life, *PC* pancreatic cancer, *PDAC* pancreatic adenocarcinoma, *MPC* metastatic pancreatic cancer/pancreatic adenocarcinoma, *LAPC* locally advanced pancreatic cancer/pancreatic adenocarcinoma, *APC* advanced pancreatic cancer/pancreatic adenocarcinoma

The mutations of tumor suppressor genes are more randomized and diverse; thus, it is very difficult to develop target therapy directly. SGT-53 is a complex of cationic liposome that could efficaciously deliver the p53 cDNA to the tumor cells. A phase II trial of SGT-53 and gemcitabine/nab-paclitaxel for MPC is ongoing (NCT02340117). Drugs targeting SMAD4 and CDKN2A are still under development.

##### Notch pathway

Notch activation triggers pancreatic nestin (+) precursor cells to accumulate and metaplastic ductal epithelium to expand. By expanding undifferentiated precursor cells, it also mediates TGF-α, which induces epithelial differentiation. A phase II study of the Notch pathway inhibitor RO4929097 was launched among MPC patients as second- or third-line treatment, and it showed a cytostatic effect rather than cell death as monotherapy [41]. A phase I study of MK0752 added to gemcitabine hydrochloride as first-line treatment in late-stage PDAC has been completed (NCT01098344). The Notch2/Notch3 antagonist tarextumab reduced the tumor-initiating cell frequency in patient-derived xenograft tumors [42]. The ALPINE trial, which studied the addition of tarextumab to nab-paclitaxel and gemcitabine, was recently completed (NCT01647828). Ponnurangam et al. found that quinomycin significantly inhibited tumor growth in nude mice, coupled with the reduction of CSC markers and Notch signaling proteins [43]. Because unwanted gastrointestinal toxicity was associated with γ-secretase inhibitors (GSIs), chemopreventive natural agents, such as curcumin, sulphoraphane, and genistein, might serve as potential Notch inhibitors while easing the toxicity [44, 45].

##### JAK/STAT pathway

The JAK family consists of JAK1, JAK2, JAK3, and TYK2, which play critical roles in hematopoiesis [46]. STAT3 is essential for disease progression in a PDAC mouse model harboring KRAS mutation [47]. High-throughput gene expression analysis found evidence of the JAK-STAT pathway in PDAC. Because phosphorylation of STAT transcription factors relies on the activation of JAK receptors, especially JAK2/STAT3, preclinical studies showed that the efficacy of JAK2 inhibitors could be predicated by phosphorylated STAT3 [47]. JAK/STAT together is related to inflammatory reaction and drives tumor progression [48, 49]. A double-blind, phase II study of the JAK1/JAK2 inhibitor ruxolitinib in combination with capecitabine for selected MPC patients with high levels of serum C-reactive protein levels showed prolonged survival and good tolerance [50]. The JANUS 1 trial is a phase III study of ruxolitinib and capecitabine as second-line therapy for MPC, but it was terminated because the results of planned interim analysis demonstrated futility (NCT02117479). Napabucasin—a STAT3 inhibitor—showed promising anti-cancer effects, as 52% of MPC patients achieved a ≥ 24-week disease control rate (DCR) in a phase Ib study (NCT02231723). A phase III study of napabucasin combined with nab-paclitaxel and gemcitabine for MPC is currently recruiting (NCT02993731).

### Therapies focused on the tumor microenvironment

The tumor microenvironment of PDAC is different from that of other solid tumors because it is rich in stroma but lacks an oxygen and blood supply. This unique environment results in disappointing drug efficacy. Thus, depleting stromal HA, enhancing drug delivery, anti-angiogenesis, and inhibiting metabolic reprogramming might prolong survival.

#### Hedgehog inhibitors

Activated pancreatic stellate cells (PSCs) are essential to neural invasion in PDAC. The overexpression of Sonic hedgehog, one of the Hedgehog (HH) signaling ligands, could activate the HH signaling pathway in PSCs, thus triggering the initiation of PDAC and resulting in desmoplastic stroma [51, 52]. In recent years, clinical efforts have been devoted to smoothened targeting, which could regulate HH signaling. The addition of vismodegib to gemcitabine experienced a setback in improving survival in MPC [53]. Vismodegib did not increase drug delivery efficacy or survival in metastatic colorectal cancer and ovarian cancer [54]. A phase II study of saridegib plus gemcitabine for MPC also announced the termination of the trial because a higher rate of progressive disease appeared in the saridegib arm. Because reducing HH signaling promotes angiogenesis, the imbalance of epithelial and stromal elements might explain the clinical trial’s failure. HH inhibitors were likely to result in a strong reduction in signaling, but they were metabolized and excreted between doses; thus, the possible key consideration to elevate the efficacy might be the HH inhibitor dosage [55]. However, there is no clinical trial concerning the combination therapy in this field. SHH and Gli1 are independent prognostic factors for resected PDAC because a lower expression of Gli1 or SHH led to longer DFS and OS [56]. Thus, HH signaling showed promise to become a diagnostic tool for APC, such as NF-κB expression, MMP9 expression, and Gli1 expression and guide therapies in the future.

#### Therapies enhancing drug delivery

PDAC is characterized by dense stroma with a redundant amount of HA, which regulates cancer initiation, progression, angiogenesis, and chemotherapy resistance. A high expression of HA is an independent prognostic factor of resected PDAC; thus, therapeutics targeting HA might cross the drug delivery barrier [57]. 4-MU is well recognized for its function to suppress HA synthesis and high safety profile, proving its anti-cancer activity in vitro and in vivo in other cancers [58, 59]. 4-MU inhibited liver metastasis in mice inoculated with human PC cells, showing the prospect for it to become a PDAC treatment targeting hyaluronan in the clinical setting.

HA interacts with CD44 on the cell surface, regulating the invasion of PDAC. The CD44-HA interaction resulted in the abrogation of cellular events by digesting HA oligosaccharides, which could be a potential direction for targeted therapy. The downstream of CA44-HA interaction could reduce metastasis to the peritoneum induced through HA by the PI3K inhibitor wortmannin [60].

Depleting stromal HA in PDAC could reduce the tumor pressure and vascular compression and improve drug delivery efficiency. A phase II study combined PEGPH20, which degrades HA, with nab-paclitaxel/gemcitabine for MPC, with promising results. The PFS was significantly higher among HA-high patients in the PEGPH20 plus nab-paclitaxel/gemcitabine arm (HR = 0.51, 95% CI 0.26–1.00, *P* = 0.048), and thromboembolic events were similar in both arms. These data suggested that PEGPH20 is a potential agent for combination therapy in the high-HA subgroup, and the results of the ongoing global phase III HALO 301 trial with co-primary endpoints are awaited (NCT01839487).

Hyaluronan also compresses the intratumoral microvasculature and creates a drug delivery barrier. Angiotensin receptor blocker (ARB) could reduce stromal collagen and hyaluronan production, downregulating the expression of TGF-β1, CCN2, and ET-1 [61]. Preclinical studies concluded that it increased vascular perfusion, oxygen, and potentiates chemotherapy [62]. A phase II clinical trial concerning FOLFIRINOX plus losartan and radiation with proton to confirm whether ARB could sensitize PDAC compared with classic chemotherapy alone is currently recruiting (NCT01821729).

#### Anti-angiogenesis

Preclinical studies have shown that vascular endothelial growth factor (VEGF) could bind to VEGFR to mediate signaling events, which increase endothelial cell proliferation and migration in PDAC. Favorable results of phase II studies did not lead to a satisfying outcome in phase III studies. A recombinant fusion protein, aflibercept, comprising VEGFR-1 and VEGFR-2, was used in a phase III randomized trial with gemcitabine for APC patients, but it was stopped for futility due to a short OS and frequent adverse events [63]. Anti-VEGF-A monoclonal antibody bevacizumab plus gemcitabine compared with gemcitabine alone in APC did not improve OS or PFS in a phase III CALGB 80303 study (mOS, 5.8 vs 5.9 months, *P* = 0.95; mPFS, 3.8 vs 2.9 months, *P* = 0.07). Similarly, sorafenib, targeting Ras-dependent signaling, and angiogenic pathways, showed considerable efficacy and safety with gemcitabine in a phase I trial. However, a randomized phase III trial—the BAYPAN study—observed no improvement in OS upon the addition of sorafenib to gemcitabine in APC [64]. A phase II study of the addition of VEGFRs, PDGFRs, and the SCFR inhibitor sunitinib to gemcitabine failed to improve PFS or OS but was associated with more toxicity [65]. Axitinib, a selective oral inhibitor of VEGFR-1, − 2 and − 3, showed an ineffective outcome in improving the survival in APC patients in phase II/III studies [66]. The contrast between preclinical studies and clinical trials may be because experimental models are rich in vascularity and lack desmoplastic reaction. The promising outcome of phase II studies was not obtained in phase III studies likely because the design of phase II studies was non-randomized and single armed. Studies of anti-angiogenic agents/gemcitabine-based therapies have achieved no superiority in survival. However, because 35% of PDACs are of the angiogenic phenotype, it is possible that a subgroup of patients would benefit from antiangiogenic therapies [67]. One hundred fifty-six patients enrolled in CALGB 80303 underwent detection for histidine-rich glycoprotein and complement factor H to predict the response to bevacizumab. However, only histidine-rich glycoprotein was weakly associated with OS [68]. Subsequently, three predictive biomarkers were discovered in the same group of patients. A low level of VEGF-D was a favorable factor to benefit from bevacizumab/gemcitabine, and Ang-2 and SDF-1 were favorable to the gemcitabine/placebo arm [69]. The identification of survival-related biomarkers and discoveries of other involved angiogenic pathways may salvage the present dilemma.

#### Metabolism

Metabolic tumor burden evaluation mainly includes total lesion glycolysis and the metabolic tumor volume, which showed strong consistency with CA19–9 and clinical prognosis for resectable PDAC [70]. Because the desmoplastic microenvironment results in hypoxia and jejuneness, PC cells metabolized 10 times more glucose than normal cells. Glucose deprivation leads to the development of mutations in genes in the KRAS pathways in PC cells, which later reprogram the cellular metabolism and sustain unrestricted tumor growth [71]. To improve survival under this nutrient-deprived condition, autophagy is activated to promote the use of an inner source [72]. Yang et al. demonstrated that chloroquine and its derivatives could effectively inhibit autophagy, leading to tumor regression in PDAC [73]. Hydroxychloroquine (HCQ) has been studied in several clinical trials as single-agent therapy in the MPC setting and as neoadjuvant therapy plus gemcitabine/nab-paclitaxel in resectable PC or MPC (NCT01978184 and NCT01506973). However, HCQ did not achieve consistent inhibition of autophagy as a monotherapy agent, and whether it is effective in combination with classic chemotherapy remains uncertain. Liang et al. demonstrated that ADP-ribosylation factor 6 (ARF6) was a downstream target of KRAS/ERK signaling. The silencing of ARF6 reduced PC cell proliferation and attenuated the Warburg effect [74].

PC cells tend to metabolize glucose through pyruvate in aerobic mitochondrial metabolism and glycolysis to convert pyruvate to lactate [75]. CPI-163—a mitochondrial metabolism inhibitor—could suppress the activity of α-ketoglutarate dehydrogenase and pyruvate dehydrogenase. A phase I study of the addition of CPI-163 to mFOLFIRINOX for MPC compared with FOLFIRINOX alone revealed that the ORR was higher in the CPI-163 arm with no increase in toxicity (ORR 53.9 vs 31.8%, NCT01835041). Argininosuccinate synthase inhibitor ADI-PEG 20 demonstrated a median PFS of 6.1 months and a median OS of 11.3 months in MPC when treated with the phase II dose combined with nab-paclitaxel and gemcitabine in a phase I/Ib study. However, adverse events increased with the use of enzyme inhibitors; thus, concern should be raised focusing on the efficacy of the combination with chemotherapy and associated toxicity profile [76].

### Immunotherapy

The immunosuppressive microenvironment of PC is highly heterogeneous and complex. Macrophages, myeloid-derived suppressor cells (MDSCs), and regulatory T cells (Tregs) are the three major leukocyte subtypes in the early stages of pancreatic intraepithelial neoplasia, which results in the rise of Tregs and inactivation of effector T cells. Immunosuppressive cells also accumulate in the peripheral blood, stroma, and PC tissues, inhibiting the proliferation and response of normal effector T cells. In addition, NK cell immunity, FoxP3 + T cells and CD8 + T cells were positively correlated with the survival of patients. Regulation of these immune cells may inhibit or limit tumor growth [77].

Immunotherapies against PDAC could be categorized as passive or active immune responses. Passive immune responses were more involved in impeding molecules that regulate cancer initiation, development, and progression. Mesothelin (MSLN) is a differentiated antigen that is overexpressed in more than 90% of PDACs. Anti-MSLN antibody SS1P binds to MSLN, resulting in the inhibition of protein synthesis and apoptosis. A phase I/II study of SS1P plus pentostatin and cyclophosphamide is being studied for PC and other MSLN-expressing malignancies (NCT01362790). Anti-MSLN monoclonal antibody MORAb-009 plus gemcitabine failed to demonstrate any clinical benefit compared with gemcitabine alone in a phase II study (mOS, 6.5 vs 6.9 months; mPFS, 3.4 vs 3.5 months; NCT00570713). Monoclonal antibodies MVT-5873 targeted at CA19–9 are under safety and tolerability study (NCT02672917). Cetuximab and matuzumab—two monoclonal antibodies that target EGFR—have demonstrated convincing preclinical results. The addition of cetuximab to gemcitabine in APC patients showed no clinical significance, while stable disease and a partial response were achieved with the addition of matuzumab to gemcitabine [23].

Active immune responses could be interpreted as exposing the tumor antigen to stimulate tumor-specific immunity. A phase I/II study of the telomerase peptide vaccine GV1001 showed a survival benefit when combined with granulocyte-macrophage colony stimulating factor (GM-CSF) in unresectable PC [78]. However, phase III studies of GV1001 plus classic chemotherapy failed to significantly prolong survival in unresectable PC compared with that in the chemotherapy-alone group (NCT00358566, TeloVac ISRCTN4382138). Pancreatic GVAX is composed of GM-CSF that is irritated by two allogenic pancreatic cancer cell lines, inducing T cells against PDAC antigens. A randomized phase II study was conducted using GVAX with low-dose cyclophosphamide (Cy) followed by CRS-207 to inhibit regulatory T cells and induce adaptive immunity [79]. The results showed that MPC patients who received Cy/GVAX followed by CRS-207 had a survival advantage compared with those who received Cy/GVAX alone. This finding is supported by Lutz ER’s finding that immune-based therapies could convert non-immunogenic tumors into immunogenic tumors. Thus, vaccine therapy plus immune-modulating agents would show better results than single-vaccine therapy [80]. Algenpantucel-L was irritated by two allogenic PC cell lines (HAPa-1, HAPa-2) and was engineered by retrovirus transduction to express galactosyltransferase. An open-label phase II trial concerning the addition of algenpantucel-L to standard adjuvant therapy for resected PDAC noted that patients benefited from 300 million cells/dose compared with 100 million cells/dose (1 year DFS, 81 vs 51%; 1 year OS, 96 vs 79%) [81]. However, the phase III IMPRESS study comparing gemcitabine plus CRT with/without algenpantucel-L for resected PC did not achieve its primary endpoint (mOS, 27.3 vs 30.4 months; NCT01072981). The poor efficacy of immunotherapy might be associated with multiple immunosuppressive mechanisms.

Bispecific antibodies (BsAb) can redirect effector cells to tumor cells without MHC restriction [82]. BsAb binds to therapeutics and tumor cells and blocks two different oncogenic mediators simultaneously [83]. The clinical outcome of BsAb is more appealing in hematologic malignancies than in solid tumors. MT110 is a bispecific T cell engager with two scFvs that bind to the CSC marker EpCAM on tumor cells and CD3 on T cells [84]. Cioffi et al. found that MT110 could eliminate CSCs of PC in vivo and in vitro [85]. Azria et al. used PC xenografts in nude mice to successfully develop a BsAb targeting TNF-α/CEA plus TNF-α and radiotherapy to control tumor growth [86]. A phase II study of BsAb targeting HER3/IGF-IR plus nab-paclitaxel and gemcitabine in MPC is currently ongoing (NCT02399137). To improve BsAb efficacy in the treatment of solid tumors, finding a specific target and extending the short half-life of BsAb are essential in future explorations.

Mycobacteria play an irreplaceable role in modulating immune responses by enhancing the crosstalk between innate and adaptive immunity. A phase II, randomized, open-label study of heat-killed mycobacterium obuense IMM-101 plus gemcitabine was well tolerated but not significantly improved in midian OS among APC patients (6.7 vs 5.6 months, *P* = 0.074) [87, 88]. However, OS was significantly improved for 2.6 months in the pre-defined metastatic subgroup in the IMM-101 plus gemcitabine group. A large, adequately powered, phase III study of IMM-101 is needed to retain clinical efficacy in treating APC.

Targeting immune checkpoint pathways are well received currently, and promising immune checkpoint inhibitors are being developed, such as CTLA-4/B7 and PD-1/PD-L1 [77]. However, 26 locally advanced and MPC patients showed no response to ipilimumab (anti-CTLA-4) except for one patient who showed a delayed response in a phase II study [89]. Pembrolizumab (anti-PD-1) demonstrated a durable response with the median PFS and OS not reached in PC deficient in mismatch repair, indicating that mutant neoantigens in mismatch repair-deficient cancers are sensitive to immune checkpoint blockade [90]. Durvalumab (anti-PD-L1) showed anti-PC activity with a 12-week DCR of 21% and an ORR of 7% in a phase II study. Additionally, MDX1105-01 (anti-PD-L1) show no objective response in 14 APC patients [91, 92]. The lack of infiltration of effector T cells might cause resistance to single-agent checkpoint inhibitor treatment in PDAC. Innovative combination therapies of immune checkpoint inhibitors are also being explored. A phase II study of MEDI4736 (anti-PD-L1) monotherapy or in combination with tremelimumab (anti-CTLA-4) in MPC was recently completed (NCT02558894). Phase I studies of durvalumab plus pexidartinib or nivolumab plus nab-paclitaxel/gemcitabine for MPC are ongoing (NCT02777710, NCT02309177). Under the treatment of ipilimumab and GVAX for previously treated PC, two patients showed stable disease (7 and 22 weeks, respectively) without a CA19–9 biochemical response in the ipilimumab arm and three patients showed prolonged disease stabilization (31, 71 and 81 weeks) with CA19–9 declined in the ipilimumab plus GVAX arm [93]. A phase II study of nivolumab (anti-PD-1 antibody) and GVAX/CRS-207 combinations was launched in MPC to improve the T cell-specific response (NCT02243371). Checkpoint blockade in combination with GVAX has the potential for clinical use and should be evaluated in larger studies. Because T cells are not the only key cell population essential to activate the immune response and immune checkpoint inhibitors, macrophages, and MDSCs are also indispensable members of tumor-infiltrating immune cells. Thus, it could be concluded that a future direction would be well-designed combinatorial immunotherapy and therapy approaches targeted at multiple immune cells.

Chimeric antigen receptors (CARs) are the core component of chimeric antigen receptor T cells (CAR-Ts), which confer T cells to tumor antigens through the recognition of MHC-independent ligands. CAR-T cell therapy is designed to redirect a patient or donor’s T cells to destroy tumor cells, which are highly targeted and show long-term persistence in vivo [94, 95]. It has achieved great success in hematologic malignancies but restrained efficacy in solid cancers because of antigen loss in tumor cells and reacting in an immune-suppressive environment that lacks exclusive antigens. Preclinical studies of EGFR-specific CAR-T cell therapy promote the antitumor activity of cytokine-induced killer cells against EGFR-positive malignancies [96]. Because EGFR is overexpressed in 90% PDAC, EGFR-specific CAR-T cell therapy presents future potent therapeutic use. HER2 overexpression leads to cellular transformation and tumorigenesis associated with a poor prognosis. However, anti-HER2-monoclonal antibodies showed no significant survival improvement in PDAC. Investigators have developed HER2-specific CAR-T cell therapy targeting HER2-positive cancer. A phase I/II study of anti-HER2 CAR-modified T cells evaluating the cytokine storm response and adverse events in refractory PDAC are currently ongoing (NCT02713984). Some studies have suggested that MSLN is the receptor of CA125/MUC16, which is closely related to metastasis in PDAC [97]. MSLN-specific CAR-T cells showed cytolytic activity toward MSLN-positive tumor cells but induce on-target/off-tumor toxicity, causing severe adverse events [98]. A phase I study of meso-CART by vascular intervention in APC is currently recruiting APC, hoping to increase the antitumor effect by suitable formation of tumor-associated antigen-targeted-CAR-T cells and decrease the accumulation of CAR-T cells in normal MSCL tissue (NCT02706782) [99]. The restricted efficacy of CAR-T cells is probably due to low CAR-T cell trafficking to tumors; thus, intravenous injection and incorporation of CARs with more effector molecules might help to improve the efficacy. Fourth-generation CARs are known to augment T cell activation and attract innate immune cells, still arousing enthusiasm for novel PDAC treatment [100].

### From bench to bedside: potential therapeutics and biomarkers

#### Cancer stem cell therapy

CSCs function as tumor-initiating cells and are prone to chemoresistance and tumor progression. The expression of aldehyde dehydrogenase in PC cells marks their stem cell features, which are associated with a poor prognosis [101]. In vivo study showed that specific CD133^+^CXCR4^+^CSCs are the only subpopulation responsible for tumor metastasis, providing a possible direction for the eradication of CSCs and target therapy [102]. The mTOR inhibitor rapamycin reduced the viability of CD133^+^ PC cells and suppressed CSC survival when combined with gemcitabine [103]. FAM83A is overexpressed significantly and associated with a poorer outcome in PDAC, which activates the well-characterized CSC-associated pathways. Silencing FAM83A inhibited TGF-β and Wnt/β-catenin pathways, resulting in oncogenicity reduction and potential as a target. Preclinical studies indicated that the JNK-ROS axis, LKB1-AMPK axis, and RAF family kinases may be involved in CSC maintenance and survival. However, because certain molecular mediators and interactions between signaling pathways remain unclear, clinical translation needs further exploration into CSC-specific pathways and markers [104].

#### MicroRNAs and long non-coding RNAs

MicroRNAs (miRNAs) are normally non-coding 22-nucleotide endogenous RNA sequences that play a key role in modulating gene expression by targeting protein-coding mRNAs and resulting in silencing and degradation. Certain miRNAs have unique predictive potential to be used in the clinic as biomarkers. Emerging data have shown that miRNAs are associated with abnormalities in transcription factors and function as both tumor suppressors and oncogenes in PDAC [105]. Sicard et al. found that miR-21 depletion prevented PDAC from progressing and the combination of miR-21 targeting and chemotherapeutic treatment resulted in tumor regression [106]. An in vivo study discovered that targeting the miR-34a delivery system could inhibit tumor growth and induce apoptosis. Such bifunctional CC9 peptide miR-34a delivery nanocomplexes are a possible future vision of effective therapy for PDAC [107]. miRNAs still require much development to be widely used in clinical settings. The plasma miR-223 level could discriminate the invasiveness between malignant IPMN and PDAC and might be applied to the early diagnosis of PDAC [108]. Additionally, miR-10b is correlated with the response to neoadjuvant therapy and is recognized as a powerful diagnostic biomarker for PDAC [109]. Schultz NA et al. identified two diagnostic miRNA panels (miR-145, miR-150, miR-223, miR-636) in whole blood that distinguished PDAC from healthy controls and potentiated the future early detection of PDAC when applied to serum CA19–9 together [110]. miRNAs (miR-21, miR-155, miR-101) were also revealed as possible non-invasive indicators for patients with the degeneration of IPMNs, fundamentally making early surgery possible and improving the prognosis. The best part of potential miRNA inhibitors compared with current approaches targeting genes and specific pathways is that they are aimed at multiple tumor suppressors in oncogenic networks simultaneously. The novel molecular targets of miRNAs that regulate molecular signaling will be identified with further research into miRNA dysregulation.

Long noncoding RNAs (lncRNAs) are non-coding RNAs with more than 200 nucleotides. Studies have shown that lncRNAs have an important function in epigenetic regulation as well as the regulation of the cell cycle and cell differentiation [111]. They function as critical regulators of processes from oncogenesis to metastasis. lncRNAs regulate EMT transition in PDAC. A novel lncRNA, LOC389641, which is upregulated in PDAC, promoted disease progression and expressed a negative correlation with E-cadherin levels. The knockdown of LOC389641 or regulation of E-cadherin could impair cell proliferation and invasion and induce apoptosis [112]. The overexpression of the lncRNA AFAP1-AS1 was found to be associated with lymph node metastasis, perineural invasion, and poor survival. It served as a prognostic marker to predict PDAC progression within 6 months and 1 year. The molecular mechanisms of lncRNAs have not been fully understood, but lncRNAs have the potential to be used as biomarkers to predict the prognosis and as rational therapeutic targets.

#### Epithelial-to-mesenchymal transition

Cells with EMT features gradually lose epithelial cell characteristic and produce mesenchymal vimentin cytoskeleton. Under certain conditions, EMT facilitates mesenchymal stem cell conversion into tumor cells. EMT enables immotile epithelial cells to acquire mesenchymal characteristic and become motile and invasive at early stage [113, 114]. Studies have shown that chemoresistance is associated with high expression of ZEB1 and vimentin in PDAC [115].

The gemcitabine-resistant cell population was observed accompanied by increased CSC markers and relocalization of EMT components like β-catenin, E-cadherin, and vimentin. CSCs and EMT-type cells have similar characteristics because they are not only both involved in the Notch and Wnt pathways but also interact with each other. Forced expression of FoxM1 promotes the expression of vimentin, Zeb-1, Snail2, and a quantitative rise in CSCs in PDAC [116]. The side population of PDAC is recognized as gemcitabine resistant because they express genes with high multidrug resistance, such as ABCG2, ABCA9, and EMT (SNAI2, LEF1). miRNAs (miR-99a, miR-100, miR-429) that gain stem cell-like properties during EMT contribute to drug resistance in PC [117]. TGF-β, TNF-α, and NF-κB signaling pathways are also closely related to the mediator of EMT [118]. TGF-β is the major regulator of EMT because it downregulates miR-200 expression and results in E-cadherin downregulation and EMT induction [119]. Although the crosstalk between TGF-β and EMT was not fully discovered, several targets have been identified to regulate EMT downstream of TGF-β for potential use in the clinic. NF-κB does not directly induce EMT, but TGF-α induces EMT through NF-κB activation. It promotes metastasis by upregulating PGE2 and EP2 signaling. Clinical trials to inhibit the TGF-β pathway (NCT01373164, NCT02734160) and Wnt pathway (NCT02050178, NCT01764477) are still ongoing.

Recent studies have demonstrated the correlation between high inflammatory activity and EMT with a bidirectional effect and joint stimulation of tumor metastasis [120]. Han et al. demonstrated that indomethacin—an inhibitor of COX-2—downregulated high-glucose-induced PC cell proliferation and invasion by regulating E-cadherin but not COX-2 [121]. They indicated that anti-inflammatory drugs could be a novel therapeutic strategy in PDAC patients who also suffered from diabetes.

### Future challenge: personalized therapies

In the past three decades, PDAC has made slow progress compared with other tumors. Classic treatments have remained gemcitabine and 5-FU-based chemo(radiation)therapy. Over the past 5 years, preclinical studies and clinical research have gradually shed light on the crosstalk between tumor cells and the complex microenvironment. Novel regimens have been developed to target PDAC oncogenesis, the tumor microenvironment, immunosuppression, signaling pathways, and DNA damage repair. Combinations of novel agents and classic chemo(radiation)therapy prolonged the survival to some extent, but the optimized treatment combinations still need validation from more high-quality clinical trials. It also highlights the importance of biomarkers associated with efficacy. Specific biomarkers assist in selecting patients with a good response and well tolerance are desperately needed, such as CA19–9, CEA, CA125, ACIN1, TNFRSCF10C, and diagnostic miRNA panels.

Dividing PDAC as four independent diseases delivers a message that different subtypes have distinct survival rate, treatment methods, and genetic characteristics. Precision treatment is based on the identification of different subtypes of PDAC, indicating that PDAC has heterogeneous and mutation-accumulated features. Because a subset of PC harbors complex rearrangement patterns with mitotic errors simultaneously rather than sequentially, it provides insight into the tumorigenesis and tumor progression for future mechanism exploration. Deep sequencing of KRAS found that a subgroup of KRAS wild-type was observed with elevated levels of mTOR pathway proteins, suggesting that mTOR inhibitors might benefit patients with KRAS wild-type PDAC. In addition, multiple KRAS mutations were detected in the same cancer cells, possibly implying that a second KRAS mutation occurred due to selective advantage and leading to stronger KRAS signaling. It is vital that we recognize these additional intratumoral KRAS mutations and associated therapeutic resistance in future clinical settings. There is no doubt that immunotherapy is the prospect of future treatment. Similar to targeted therapy, it is crucial that further investigations should be made to characterize the underlying mechanisms among patients with the immunogenic subtype, and clinical trials of combinatorial immunotherapies are encouraged after certain patient selection.

## Conclusions

With the success of the JASPAC-01 trial, S-1 paved its way as monotherapy for patients with resected PDAC after surgery or MPC patients with poor performance. Anti-PD-1 antibody pembrolizumab also showed promising outcomes in highly selective patients. Some combinations of novel agents may be efficacious as monotherapy, such as the nanoliposome irinotecan prior to 5-FU/LV, the HA degrader PEGPH20, or STAT3 inhibitor napabucasin in combination with nab-paclitaxel/gemcitabine, the MEK1/2 inhibitor refametinib in combination with gemcitabine, and the immune checkpoint inhibitor ipilimumab in combination with GVAX. It is still hopeful for some rational combinations of novel agents to produce a certain clinical benefit, encouraging patients to enroll in clinical trials that would undoubtedly accelerate the process of improving outcomes for this fatal malignancy.

## Abbreviations

5-FU: 5-fluorouracil
APC: Advanced pancreatic cancer
ARB: Angiotensin receptor blocker
ARF6: ADP-ribosylation factor 6
BsAb: Bispecific antibodies
CARs: Chimeric antigen receptors
CAR-Ts: Chimeric antigen receptor T cells
CSCs: Cancer stem cells
CTLA-4: Cytotoxic T lymphocyte-associated antigen 4
DCR: Disease control rate
DFS: Disease-free survival
EGFR: Epidermal growth factor receptors
EMT: Epithelial-to-mesenchymal transition
FOLFIRINOX: Folinic acid, fluorouracil, irinotecan and oxaliplatin
GM-CSF: Granulocyte-macrophage colony stimulating factor
GSIs: γ-Secretase inhibitors
HA: Hyaluronic acid
HCQ: Hydroxychloroquine
HER2: Human epidermal growth factor receptor 2
HH: Hedgehog
ILK: Integrin-linked kinase
lncRNA: Long noncoding RNA
LV: Leucovorin
MDSCs: Myeloid-derived suppressor cells
MEK: Mitogen/extracellular signal-related kinase
miRNAs: MicroRNAs
MPC: Metastatic pancreatic cancer/pancreatic ductal adenocarcinoma
MSLN: Mesothelin
ORR: Objective response rate
OS: Overall survival
PC: Pancreatic cancer
PD-1: Programmed cell death protein 1
PDAC: Pancreatic ductal adenocarcinoma
PEGPH20: PEGylated human recombinant PH20 hyaluronidase
PFS: Progression-free survival
PI3K/AKT/mTOR: Phosphatidylinositol 3 kinase/protein kinase B, PKB/mammalian target of rapamycin
PSCs: Pancreatic stellate cells
TGFβ: Transforming growth factor β
Tregs: Regulatory T cells
VEGF: Vascular endothelial growth factor
VEGFR: Vascular endothelial growth factor receptor
